# Serious fungal infections in Chile

**DOI:** 10.1007/s10096-017-2925-8

**Published:** 2017-02-10

**Authors:** E. Alvarez Duarte, D. W. Denning

**Affiliations:** 1 Mycology Unit—Filamentous Fungal Section, Biomedical Sciences Department, University of Chile, Santiago, Chile; 2 University of Manchester, Manchester Academic Health Science Centre and National Aspergillosis Centre, University Hospital of South Manchester, Manchester, UK

## Abstract

The incidence and prevalence of fungal infections in Chile are unknown. Here, we have estimated the burden of serious fungal diseases from data obtained from clinical reports, WHO reports, Chilean census, OECD reports and comprehensive literature search available on PubMed and SciELO, among other scientific resources. Due the lack of official data about fungal diseases, frequencies were calculated based on the specific populations at risk. Recurrent vulvovaginal candidiasis (>4 episodes/year) is estimated to occur in 3108/100,000. Using a low international average rate of 5/100,000, we estimate 878 candidaemia cases and 132 patients with intra-abdominal candidiasis. Due to the low incidence of pulmonary tuberculosis (TB) in Chile, limited numbers of patients with chronic pulmonary aspergillosis are likely: a total of 1212, 25% following TB. Invasive aspergillosis is estimated to affect 296 patients following leukaemia therapy, transplantation and chronic obstructive pulmonary disease (COPD), 1.7/100,000. In addition, allergic bronchopulmonary aspergillosis (ABPA) and severe asthma with fungal sensitisation (SAFS) were estimated to be around 97.9/100,000 and 127/100,000 respectively, in 675,772 adult asthmatics and 1700 CF patients. Given a 38,000 human immunodeficiency virus (HIV) population, with around 2189 new cases of acquired immune deficiency syndrome (AIDS) annually, cryptococcal meningitis and *Pneumocystis* pneumonia are estimated at 0.12/100,000 and 4.3/100,000, respectively. In total, 325,000 (1.9%) people in Chile develop serious fungal infections annually. Respiratory fungal disease predominates in Chile; a national action plan for fungal disease is urgently needed, including epidemiological studies to validate the estimates.

## Introduction

The incidence of invasive fungal disease (IFD) as an important cause of morbidity and mortality has increased dramatically in recent years, particularly in immunocompromised patients [1]. Mortality associated with fungal diseases is substantially higher than those reported for most viral or bacterial diseases. Over 1 million people every year die due a fungal infection [2]. Additionally, several infectious processes in immunocompetent people caused by unusual opportunistic fungi have been described in recent years [3–5].

The most frequent opportunistic, ubiquitous and cosmopolitan infections are candidiasis (caused by *Candida* species), aspergillosis (caused by *Aspergillus* species) and mucormycosis (caused by species of the Mucorales order) [6]. Among the endemic mycoses such as histoplasmosis, blastomycosis, coccidioidomycosis and paracoccidioidomycosis, no autochthonous cases have been described in Chile [7], except for tourists or immigrants.

In Chile, studies conducted to investigate the epidemiology, evolution, genetics and therapeutic management of fungal infections caused by opportunistic fungi are scarce. No comprehensive studies have been conducted on antifungal resistance frequency or mechanisms including emerging species involved in mycosis. Moreover, data about the burden of invasive fungal diseases is limited due to the lack of surveillance programmes. The aim of this work was to assemble all available data from reliable sources and estimate the incidence and prevalence of fungal diseases in Chile.

## Methods

In order to elucidate the serious fungal burden in Chile, we performed an exhaustive search of epidemiological reports with unsuccessful results. In order to estimate the fungal burden, data derived from the Organisation for Economic Co-operation and Development (OECD), World Health Organization (WHO), the Joint United Nations Programme on HIV/AIDS (UNAIDS), Chilean Ministry of Health (AUGE Clinical Guides), the Public Health Institute of Chile and local reports were used in the present study. When no data existed, risk populations were used to estimate frequencies of fungal infections, using the previously described methodology by LIFE (http://www.LIFE-worldwide.org).

## Results and discussion

Chile is a South American country, with a population of approximately 17.5 million, 23% being children under 15 years old and 16% being women >60 years old (http://data.un.org/CountryProfile.aspx?crName=chile; https://data.oecd.org/chile.htm). The 2013 GDP was $15,732. No epidemiological reports were obtained from any of the databases such as PubMed, MEDLINE, MedFacts etc. Estimated numbers and rates of key serious fungal infections per year are shown in Table 1.

**Table 1:** Estimated burden of fungal disease in Chile

Infection	Number of infections per underlying disorder per year					Total burden	Rate/100,000
	None	HIV/AIDS	Respiratory	Cancer/Tx	ICU		
Oesophageal candidiasis	–	2650	–	–	–	2650	15
Oral candidiasis	–	6750	–	–	–	6750	38
Candidaemia	–	–	–	614	264	878	5
*Candida* peritonitis	–	–	–	–	132	132	0.8
Recurrent vaginal candidiasis (>4 episodes per year)	272,812	–	–	–	–	272,812	3108
ABPA	–	–	17,183	–	–	17,183	97.9
SAFS	–	–	22,300	–	–	22,300	127
Chronic pulmonary aspergillosis	–	–	1212	–	–	1212	6.90
Invasive aspergillosis	–	–	–	137	159	296	1.7
Cryptococcal meningitis	–	22	–	–	–	22	0.12
*Pneumocystis* pneumonia	–	766	–	–	–	766	4.3
Mucormycosis	–	–	–	–	–	35	0.2
Total burden estimated						∼325,036	

*ABPA* Allergic bronchopulmonary aspergillosis, *SAFS* Severe asthma with fungal sensitisation

### *Candida* infections

Recurrent vulvovaginal candidiasis (>4 episodes/year) is estimated to occur in ∼272,812 women (3108/100,000) between the ages of 15 and 50 years. Using a low international average rate of 5/100,000, we estimate 878 episodes of candidaemia, including nearly 132 patients with intra-abdominal candidiasis. Unfortunately, and despite their low frequency, these are associated with poor outcomes and high hospitalisation costs due the prolonged stay plus antifungal treatments [8]. Also, the incidence estimated in the present report constitutes the lowest in Latin America, but as with other countries, is an under-estimate of burden because blood culture is only ∼40% sensitive for the diagnosis of invasive candidiasis [9, 10] and worse still for intra-abdominal candidiasis [11, 12]. The low rate of candidaemia from Chile contrasts with those reported from other South American countries [13]. A surveillance national programme for candidaemia started 2 years ago, so additional data are likely to emerge soon. Based on our laboratory data, the species most prevalent in candidaemia are *Candida albicans*, followed by *C. parapsilosis* complex and *C. glabrata*/*tropicalis* (unpublished results). In this way, our results agree with those recently published from Brazil [14]. Also, a recent outbreak due to *Candida parapsilosis* complex was observed in Chile, affecting mainly adults on haemodialysis in ten centres of our country [15].

Based on the at-risk population, oesophageal and oral candidiasis were estimated in a total of around 2650 and 6750 cases, respectively; we have not been able to estimate the numbers in cancer and other conditions.

### Aspergillosis and severe asthma

Invasive aspergillosis (IA) was estimated in haematological malignancy, after transplantation and in chronic obstructive pulmonary disease (COPD) patients admitted to hospital as being about ∼1.7/100,000. Using a population rate of 3/100,000 for acute myeloid leukaemia (527 patients a year) and an annual incidence of 10% in this patient group and an equal number of IA cases in all other leukaemia and lymphoma patients, we estimated 137 IA cases in these populations. In addition, we found that there were ∼200 allogeneic haematological stem cell transplant recipients, 270 renal, 22 lung, 21 heart and 85 liver transplants in 2014. These patients contribute an additional 33 IA patients annually, assuming attack rates of 10, 1, 20, 6 and 4%, respectively. COPD is estimated to affect 16.9% of the population over 40 years [16, 17]. Using the rate of IA of 1.3% of admissions to hospital in Madrid, this predicts 159 patients annually with IA in COPD patients, many in intensive care [18]. There are an estimated 2989 patients with lung cancer in 2012 (Globoscan), and assuming that IA occurs in 2.6% of cases [19], an additional 82 IA cases would be anticipated annually.

Due the low incidence of pulmonary tuberculosis (TB) in Chile (∼2185 cases in 2014, ∼8.9% in HIV-positive patients) [20], we estimate 96 new chronic pulmonary aspergillosis patients after TB each year and a 5-year period prevalence of 303 patients. The proportion of all chronic pulmonary aspergillosis patients prior to TB varies by the frequency of TB, and given the high rate of COPD and moderate rate of asthma in Chile, we have used a 25% proportion attributable to TB. This gives a total estimated prevalence of chronic pulmonary aspergillosis of 1212 patients (6.9/100,000).

Approximately 5% of the adult Chilean population has asthma [21]. Of these, about 10% probably have severe asthma. If we assume that a conservative 33% have fungal sensitisation [22, 23], then ∼22,300 patients with have severe asthma with fungal sensitisation (SAFS) (127/100,000). Overall sensitisation rates in asthma to *Alternaria* are documented in Chile [24], but not to allergenic fungi, in severe asthma. Allergic bronchopulmonary aspergillosis (ABPA) affects about 2.5% of the 675,772 adult asthmatic patients, and is rare in children, so 17,183 probably have ABPA. There is probably some overlap between these groups as some patients with ABPA have severe asthma and all are sensitised to *Aspergillus*. Based on our data, the incidence and prevalence of respiratory fungal infections are one of the lowest in Latin American, contrasting with those reported for Brazil [25] and the Dominican Republic [26]. However, the lack of a prospective cohort study of ABPA or SAFS in Latin America or Chile, and no fungal sensitisation data apart from *Alternaria* spp., is a major limiting factor for our estimates.

### Other infections

Given a 38,000 human immunodeficiency virus (HIV) population, with around to 2189 new cases of acquired immune deficiency syndrome (AIDS) annually, cryptococcal meningitis and *Pneumocystis* pneumonia (PCP) are estimated at 0.12/100,000 and 4.3/100,000, respectively. This is based on a rate of 1% of new cases presenting with cryptococcal meningitis and 35% presenting with PCP. We have not been able to estimate PCP cases in non-HIV patients, although it is likely to be similar to those in HIV, based on our experience and documentation in other countries. These estimates were also among the lowest in South America, which contrasts with those reported for Brazil (4.7/100,000 and 3.52/100,000, respectively) [25] and Trinidad and Tobago (30/100,000 and 3.7/100,000, respectively) [27].

Other mycoses such as endemic fungal diseases were not considered due the lack of autochthonous and confirmed cases. In one local publication, Wolff reported an allochthonous outbreak of histoplasmosis in a group of Chilean travellers to the Ecuador jungle [28]. If the rate of mucormycosis is 0.2/100,000, we would expect 35 cases annually, primarily in diabetic and immunocompromised patients.

We know from clinical experience that superficial fungal infections of skin and mucosa are the most frequent mycoses in Chile (tinea unguium, tinea pedis and tinea capitis). These are primarily related to dermatophytes, followed by *Candida* spp. and *Malassezia* spp. In the dermatophytes group, the most prevalent aetiological agents are *Trichophyton rubrum*, followed by *T. mentagrophytes* and *Microsporum canis*. Other *Trichophyton* species, such as *T. tonsurans*, are very rare and mainly associated with tinea capitis in immigrants (unpublished data, available in our Lab. Databank). There are no data on fungal keratitis or tinea capitis, although cases are seen in Chile.

## Conclusions

The present study indicates that around 1.9% (∼325,000) of the Chilean population is affected by serious fungal infections. However, the estimation of fungal diseases presented in this study may be under- (or over-)estimated. These estimates have multiple limitations, the most important of which is a dearth of local data on actual fungal disease rates in at-risk populations. Another significant limitation is the incomplete nature of the estimates: no data on fungal rhinosinusitis, on *Pneumocystis* pneumonia (PCP) in non-human immunodeficiency virus (HIV) patients, on oral and oesophageal candidiasis in non-HIV patients and others are available. Even when such data are collected, routine clinical care underestimates the incidence and prevalence of fungal disease because the key diagnostic test(s) is(are) not requested by unsuspecting clinicians. Most key non-culture diagnostic tests are available in Chile but not always utilised to the maximum extent. In addition, data about fungal diseases came mainly from central areas of our ‘long’ country. No data on clinical cases, epidemiology and reports are available from remote regions, contributing to the lack of knowledge about the actual serious fungal disease burden.

To our knowledge, this study represents the first attempt at a comprehensive estimation of the burden of fungal disease in Chile. Hopefully, this rough and preliminary national burden estimate will stimulate some epidemiological studies to better document the actual burden of fungal diseases.

Further epidemiological studies are clearly needed to validate and extend these estimates. This study is part of a worldwide initiative lead by LIFE (http://www.life-worldwide.org/), which has the aim of calculating the global fungal burden in order to establish the importance of mycoses in public health around the world.
